# Survivorship in robotic total knee arthroplasty compared with conventional total knee arthroplasty: A systematic review and meta-analysis

**DOI:** 10.1186/s42836-025-00304-3

**Published:** 2025-04-08

**Authors:** Jiawei Chen, Ryan Wai Keong Loke, Katelyn Kaye-Ling Lim, Barry Wei Loong Tan

**Affiliations:** 1 National University of Singapore, Yong Loo Lin School of Medicine, National University Health System, Singapore, Singapore; 2 Department of Orthopaedics, National University Hospital, National University Health System, Singapore, Singapore

**Keywords:** Total knee replacement, Arthroplasty, Robotic; Conventional, Meta-analysis

## Abstract

**Background:**

Total knee arthroplasty (TKA) is the gold standard surgical management for end-stage knee osteoarthritis (OA). Robotic TKA (rTKA) was developed to improve bone preparation accuracy and increase reproducibility. In many settings internationally, rTKA systems have significantly higher costs for patients, and survivorship outcomes are unclear. There are several prior meta-analyses, but these focused on clinical and radiologic outcomes, and to our knowledge, none have evaluated survival. Differences in survival between semi-active or active robotic systems are also not well investigated.

**Study Design:**

Meta-analysis.

**Methods:**

A random-effects meta-analysis was conducted on comparative studies between robotic-assisted TKAs and conventional TKAs (cTKAs) in patients undergoing TKA for primary knee OA. We searched MEDLINE, Embase, Cochrane Library, and SCOPUS from inception to 19 December 2024. Outcomes assessed were the implant survival in robotic-assisted TKA compared to conventional methods in standard primary knee OA cases, with subgrouping between active and semi-active systems performed. Secondary outcomes included associated complications, post-operative pain scores, and functional outcomes.

**Results:**

A total of 20 comparative studies were included in the meta-analysis. Among them, 2,804 patients underwent cTKA, while 2,599 underwent rTKA. At two years, the pooled survivorship rate was 97.9% (95% CI: 96–99) in the conventional group and 98.3% (95% CI: 96.2–99.2) in the robotic group. There were no significant differences between the groups (*P* = 0.7). There were no significant differences between the robotic (semi-active) group and the conventional group (*P* = 0.5) on further unpaired T-Testing.

Between 2 and 5 years, pooled survivorship rates in the conventional group were 96.8% (95% CI: 90.3–99) and 97.1% (95% CI: 91.3–99) in the robotic group. There were no significant differences between groups (*P* = 0.9). At ten years postoperatively, pooled survivorship rates in the conventional group were 96.9% (95% CI: 95–98) and 97.8% (95% CI: 96.7–98.5) in the robotic group. There were no significant differences between the groups (*P* = 0.3).

**Conclusion:**

Conventional TKA is non-inferior to rTKA at short and long-term follow-up with regard to implant survival, complications, and postoperative pain scores, while rTKA shows subtle improvements in functional outcome measures.

**Trial registration:**

CRD42024540997.

## Introduction

Knee osteoarthritis (OA) is a type of chronic degenerative arthritis resulting in cartilage degeneration, bony erosions, osteophyte formation, and joint inflammation—resulting in loss of function [1, 2]. Over 86 million individuals aged 20 and above suffer from knee OA globally, and its prevalence among younger groups is on the rise [3, 4]. The gold standard for advanced OA is total knee arthroplasty (TKA) or unicompartmental knee arthroplasty (UKA) in isolated medial or lateral knee OA.[5–7].

Robotic TKA (rTKA) was developed to improve bone preparation accuracy and increase reproducibility [8, 9]. Accuracy in implant positioning enables achievement of surgeon-targeted flexion and extension gaps, allowing for functional alignment without soft tissue releases. It has demonstrated improved patient-reported outcome scores compared with mechanical or kinematic alignment in conventional TKA (cTKA) [10–13]. Robots are either “active,” such as the ACROBOT® or ROBODOC® systems where the robotic arm moves autonomously after the surgeon does preoperative planning, or “semi-active,” where the bone cuts are still made by the surgeon while robotic assistance ensures the cuts are accurate to the preoperative plan. Four major semi-active robotic systems are in use presently: ROSA® by Zimmer-Biomet, MAKO® by Stryker, CORI™ by Smith & Nephew, and VELYS™ by Johnson & Johnson [9].

Robotic TKA systems have significantly higher costs for patients in many settings internationally, and survivorship outcomes are unclear. For active systems, despite better alignment accuracy, concerns over complications and limited improvement in clinical outcomes may have deterred long-term use and hence resulted in fewer studies in literature [14]. Semi-active systems were only launched recently—the MAKO® knee system was launched in 2017, followed by ROSA® in 2018 and CORI™ & VELYS™ shortly after [15]. The COVID-19 pandemic also led to a large decline in arthroplasty surgeries, which possibly affected longer-term data collection and assessments [16]. There are several prior meta-analyses, but these focused on clinical and radiologic outcomes, and to our knowledge, none have evaluated survival [17–22]. Operational differences between active and semi-active systems also underscore the need to evaluate whether these distinctions translate into clinically meaningful differences in outcomes. This study primarily assessed implant survival in rTKA compared to conventional methods in standard primary knee OA cases—complex cases may reveal the utility of robotics more clearly, but the paucity of data on the aforementioned outcomes in standard cases should be tackled first. Subgrouping between active and semi-active systems was also performed. Secondary outcomes included cost-effectiveness and associated complications. We hypothesised that with improved implant positioning accuracy and lesser soft tissue dissection, rTKAs will yield improved survivorship, fewer complications, and cost-effectiveness.

## Methods

### Data sources and search strategy

This study was conducted in adherence with the PRISMA (Preferred Reporting Items for Systematic reviews and Meta-analyses Statement) guidelines [23]. The protocol for this systematic review and meta-analysis was registered with the PROSPERO (ID: CRD422024540997) International prospective register of systematic reviews. We searched electronic databases MEDLINE, Embase, Cochrane Library, and SCOPUS from inception to December 19, 2024, for relevant studies using keywords and terms synonymous with robotic and conventional TKAs in patients with primary knee OA and respective survival outcomes and complications. We did not limit our search to only articles written in the English language. Our search strategy can be found in the Supplementary Material.

### Study selection

Comparative studies reporting on the survival outcomes of rTKA and cTKA and associated complications were included in our meta-analysis. Studies were selected a priori based on the study population, intervention, outcomes measured, and study design (Table 1). Studies were included if they met the following criteria: (1) comparative studies evaluating robotic versus conventional TKA; (2) studies involving patients undergoing TKA for primary OA; (3) prospective or retrospective clinical studies, including randomized controlled trials (RCTs); and (4) studies reporting survivorship and complication outcomes.

**Table 1:** Inclusion and exclusion criteria

Inclusion Criteria	Exclusion Criteria
Comparative studies between robotic and conventional TKA	Case reports, review articles, editorials, technical notes, commentaries
Patients undergoing TKA for primary OA	Patients undergoing TKA for conditions other than primary OA, such as rheumatoid arthritis or trauma
Prospective/retrospective clinical studies and RCTs	Complex TKA cases such as revision TKA or conversion TKA from prior high tibial osteotomy
Survivorship & complication outcomes Studies with no missing or incomplete data with respect to key demographics and outcomes	Nonclinical, in-vitro, biomechanical studies, animal or cadaveric studies Studies with assessed severe or critical risk-of-bias, missing or incomplete data

Studies were excluded if they consisted of case reports, review articles, editorials, technical notes, or commentaries. Additionally, studies were excluded if they involved: (1) patients undergoing TKA for conditions other than primary OA (e.g., rheumatoid arthritis or trauma); (2) complex TKA cases such as revision TKA or conversion from high tibial osteotomy; (3) nonclinical studies, including in-vitro, biomechanical, animal, or cadaveric research; and (4) studies assessed as having a severe or critical risk of bias. Patients undergoing prior procedures before rTKA was not seen as a criterion for exclusion of an article; however, studies with assessed severe or critical risk-of-bias were deemed a factor for exclusion. Surgeon experience is a documented factor that affects TKA outcomes, but for this study, all performing surgeons were experienced and operating in high-volume centres.

The inclusion of an article was evaluated by three independent blinded authors (R.L., J.W., and K.L.), with any abstentions being resolved by the senior author (B.T). Inter-reviewer agreement was assessed with Cohen’s kappa statistic.

### Risk of bias and quality assessment

The same three researchers independently assessed the risk of bias of the included studies. Quality assessment of non-randomized articles was performed using the ROBINS-I tool, which grades each article on seven domains [24]. For RCTs, the Cochrane risk-of-bias tool was used. A summary of the risk of bias and quality assessment of included studies can be found within the Supplementary Material. Studies with severe or critical risk-of-bias were deemed not suitable for inclusion in the present study. Further subgrouping between retrospective and prospective study designs was also performed.

### Data extraction and outcomes

Data was extracted from the included studies by the same three researchers independently, and any discrepancies were resolved by the senior author subsequently. Data extraction was performed to extract basic study characteristics (first author, year of publication, study design, level of evidence, average age of patients, sample size, follow-up duration, proportion by gender, surgical duration, and time to surgery). Primary outcomes considered for this study were survival outcomes (survival rates and reasons for revision). It has been reported that the aetiologies for TKA revisions differ at 2-year, 5-year, and 10-year postoperatively. Therefore, these postoperative timeframes were used in this study to investigate which revision aetiology might be implicated when rTKA is used compared to cTKA. As secondary outcomes, overall complication rates, specific complications (infection, periprosthetic fracture, aseptic loosening, pain, stifness etc.), post-operative pain scores and functional outcome scores such as the Knee Society Score (KSS)—knee and function scores and the Western Ontario and McMaster Universities Osteoarthritis Index (WOMAC) score were noted.

Means and standard deviations (SD) were extracted for the pooling of continuous outcome data. When means and SD were unavailable and instead data were presented as medians with ranges, we derived the means and SD in accordance with Wan and colleagues (2014) [25]. Binary outcome data were extracted in the form of the number of events that occurred per sample size.

### Statistical analysis

Statistical analyses were performed using RStudio (Version 2022.12.0 + 353, Posit, PBC, Boston, MA, USA). We performed a random-effects (Dersimonian-and-Laird) meta-analysis to synthesize continuous and binary outcomes using the respective *metamean* and *metaprop* functions of the R meta package.

Continuous outcomes were pooled using the weighted mean approach with random effects, and the Dersimonian-and-Laird (DL) estimator was applied for between-study variance. Meta-analyses of proportions were conducted for binary outcomes, using random effects modelling. The lower and upper confidence limits for the 95% confidence intervals were estimated using the Clopper-Pearson method and the DL estimator applied for between-study variance.* P*-value was calculated directly based on the estimated proportions and their standard errors using the *Z*-test.

We assessed statistical heterogeneity among studies by visual inspection of forest plots, as well as *I*^*2*^ and *τ*^2^. *I*^*2*^ values of 25%, 50%, and 75% were thresholds for low, moderate, or high heterogeneity, respectively.

We performed prespecified subgroup analyses for each study design (prospective or retrospective) and risk of bias (low, moderate, serious, or critical). Further sensitivity analysis was to be done on studies with serious risk of bias to assess suitability for inclusion. Studies with a critical risk of bias would not be included.

Further subgrouping between active or semi-active systems was also done. Publication bias was assessed by the visual inspection of the funnel plots and Egger’s test.

## Results

### Summary of included articles

**Fig. 1:**
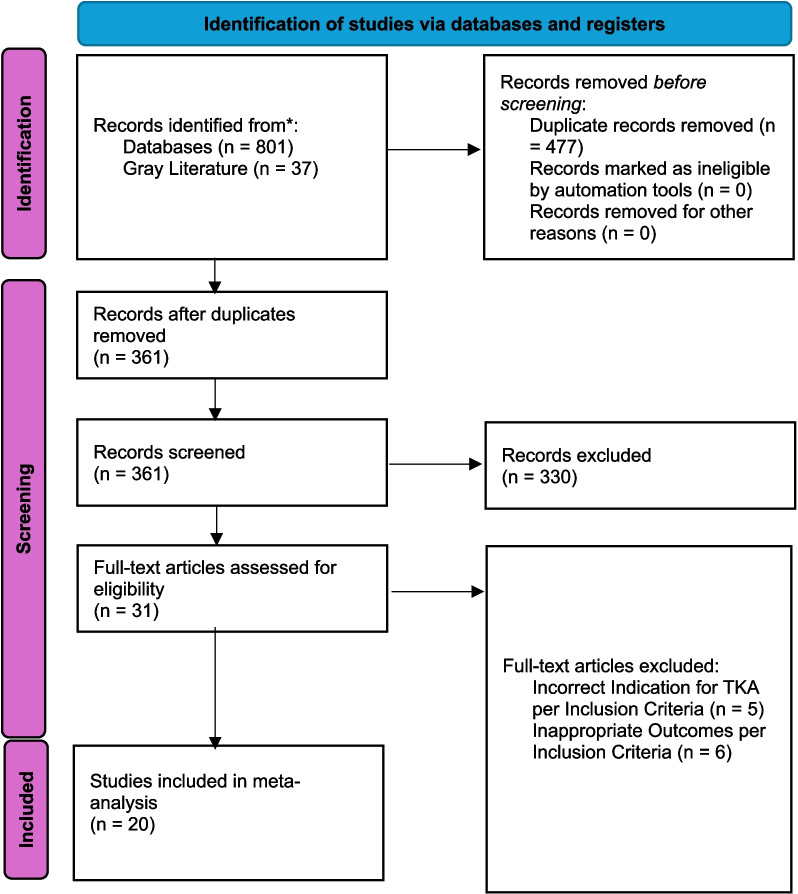
PRISMA schema

A systematic search of the available literature was done using our search strategy. It yielded a total of 838 studies. After removing 477 duplicate records, the remaining 361 studies underwent a detailed title and abstract screening. A total of 330 studies were then excluded after screening, leaving 31 full-text articles to undergo full-text review. From the full-text articles, 11 were excluded for the following reasons: Incorrect TKA indication per inclusion criteria (*n* = 5), incorrect outcomes per inclusion criteria (*n* = 6), and 20 articles fit the inclusion criteria and were thus considered for review (Fig. 1).

Of the included studies, 12 had a retrospective study design, and 8 had a prospective study design. The ROBINS-I tool was used to ascertain the risk-of-bias and quality of the non-randomized papers, while the Cochrane Risk-of-Bias tool was used for RCTs. The majority of the studies were classified as low or moderate risk of bias. None were deemed serious risk-of-bias.

**Fig. 2:**
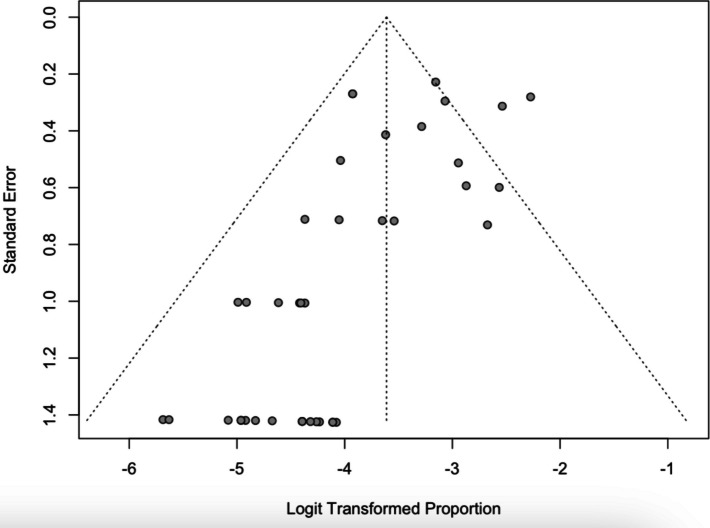
Funnel plot of overall survivorship

Publication bias was assessed based on visual analysis of funnel plot and Egger’s test based on our primary outcome—overall survivorship rates. The relatively symmetrical funnel plot (Fig. 2) and Egger’s test (*P* = 0.89) suggest no significant publication bias is present. A summary of the details of the included studies are found in Appendix A, while the risk-of-bias, quality assessment, and sensitivity analyses can be found in the Supplementary material.

### Patient characteristics

A total of 2,804 patients underwent cTKA, while 2,599 underwent rTKA. Baseline demographic characteristics of patients included in our study are shown in Table 2.

**Table 2:** Baseline demographics

	**Sample Size, *n***	**Age, y**	**BMI, kg m**^**−2**^	**Men (%)**	**Surgical Duration (mins)**
Robotic TKA	2,599	67.56 (67.00–68.12)	29.34 (28.86–29.82)	28.3	116.81 (107.60–126.01)
Conventional TKA	2,804	67.67 (66.88–68.45)	29.68 (29.19–30.17)	28.0	91.65 (87.06–96.24)

### Survival outcomes

A summary, including the study title, revisions, and reasons for revisions, can be found in Table 3.

**Table 3:** Summary of revisions and reason(s) for revisions

Author	Follow Up, m	Robot	Revisions	Total Knees	Reason(s) for Revision
Adamska 2023[26]	12 ± 0.1	Semi Active: NAVIO CORI™	cTKA: 0 rTKA: 0	cTKA: 68 (68) rTKA: 147 (147)	
Albelooshi 2023[27]	24 ± 0.1	Semi Active: NAVIO	cTKA: 0 rTKA: 2	cTKA: 34 (34) rTKA: 102 (102)	rTKA Post-traumatic femur Fracture (1) Soft Tissue Impingement (2)
Bolam 2022[28]	21.3 ± 9	Semi Active: ROSA®	cTKA: 1 rTKA: 0	cTKA: 80 (83) rTKA: 52 (53)	cTKA Mid-flexion Instability (1)
Boucher 2022[29]	24 ± 0	Semi Active: ROSA®	cTKA: 1 rTKA: 1	cTKA: 137 (137) rTKA: 155 (160)	cTKA Instability (1) rTKA Instability and Extensor Mechanism Failure (1)
Cho 2018[30]	cTKA: 134.4 ± 13.2 rTKA: 129.6 ± 10.8	Active: ROBODOC®	cTKA: 6 rTKA: 2	cTKA: 196 (230) rTKA: 155 (160)	cTKA Infection (1), PE Wear (2), Aseptic Loosening (2), Instability (1) rTKA Infection (2)
De Grave 2023[31]	12 ± 0.1	Semi Active: MAKO®	cTKA: 0 rTKA: 0	cTKA: 40 (40) rTKA: 80 (80)	
Jeon 2019[32]	cTKA: 129.5 ± 9.9 rTKA: 128.7 ± 7.9	Active: ROBODOC®	cTKA: 2 rTKA: 1	cTKA: 54 (79) rTKA: 78 (84)	cTKA Aseptic Loosening (2) rTKA Aseptic Loosening (1)
Kenanidis 2023[33]	6 ± 0.1	Semi Active: ROSA®	cTKA: 0 rTKA: 0	cTKA: 30 (30) rTKA: 30 (30)	
Kim 2019[34]	cTKA: 168 ± 15 rTKA: 156 ± 15	Active: ROBODOC®	cTKA: 14 rTKA: 14	cTKA: 674 (724) rTKA: 674 (724)	NR
Lau 2023[35]	12 ± 0.1	Semi Active: NAVIO CORI™	cTKA: 0 rTKA: 0	cTKA: 71 (71) rTKA: 71 (71)	
Lee 2023[36]	cTKA: 141.6 ± 18 rTKA: 142.8 ± 18	Active: ROBODOC®	cTKA: 12 rTKA: 7	cTKA: 270 (270) rTKA: 194 (194)	cTKA Aseptic Loosening (11), PE Wear (1) rTKA Aseptic Loosening (5), Infection (2)
Liow 2016[37]	24 ± 0.1	Active: ROBODOC®	cTKA: 0 rTKA: 2	cTKA: 29 (29) rTKA: 31 (31)	rTKA Persistent Lateral Side Knee Pain (2)
Lychagin 2023[38]	36 ± 0	Active: TSOLUTION ONE	cTKA: 0 rTKA: 3	cTKA: 62 (62) rTKA: 56 (56)	rTKA Aseptic Loosening (2), Infection (1)
Marchand 2023[39]	24 ± 0.1	Semi Active: MAKO®	cTKA: 4 rTKA: 1	cTKA: 80 (80) rTKA: 80 (80)	cTKA Component Malposition (2), Hemarthrosis (1), Tibial Loosening (1) rTKA Stiffness (1)
Marsawa 2022[40]	24 ± 0	Semi Active: NAVIO	cTKA: 11 rTKA: 14	cTKA: 150 (150) rTKA: 150 (150)	cTKA Joint Instability and Pain (11) rTKA Joint Instability and Pain (14)
Mitchell 2021[41]	12 ± 0	Semi Active: MAKO®	cTKA: 0 rTKA: 1	cTKA: 139 (139) rTKA: 148 (148))	NR
Naziri 2019[42]	12 ± 0	Semi Active: NAVIO CORI™	cTKA: 0 rTKA: 0	cTKA: 40 (40) rTKA: 40 (40)	
Vandenberk 2023[43]	cTKA: 29.7 ± 0.1 rTKA: 31.1 ± 0	Semi Active: NAVIO	cTKA: 20 rTKA: 4	cTKA: 489 (489) rTKA: 231 (231)	cTKA ITB release for friction (4); Fracture (4); DAIR (2); 5 two stage revision; open arthrolysis (1); resection of lateral osteophyte (1); Removal of perforating ACL screw (1) rTKA ITB release for friction (1); Fracture (2) Arthroscopic plica resection (1)
Xu 2022[44]	3 ± 0	Semi Active: YUANHUA-TKA	cTKA: 0 rTKA: 0	cTKA: 35 (35) rTKA: 37 (37)	
Yang 2017[45]	cTKA: 121.2 ± 11.1 rTKA: 126 ± 9.3	Active: ROBODOC®	cTKA: 3 rTKA: 2	cTKA: 42 (42) rTKA: 71 (71)	cTKA Infection (2), wear of the polyethylene liner (1) rTKA periprosthetic joint infections (2)

#### Two-year survival

**Fig. 3:**
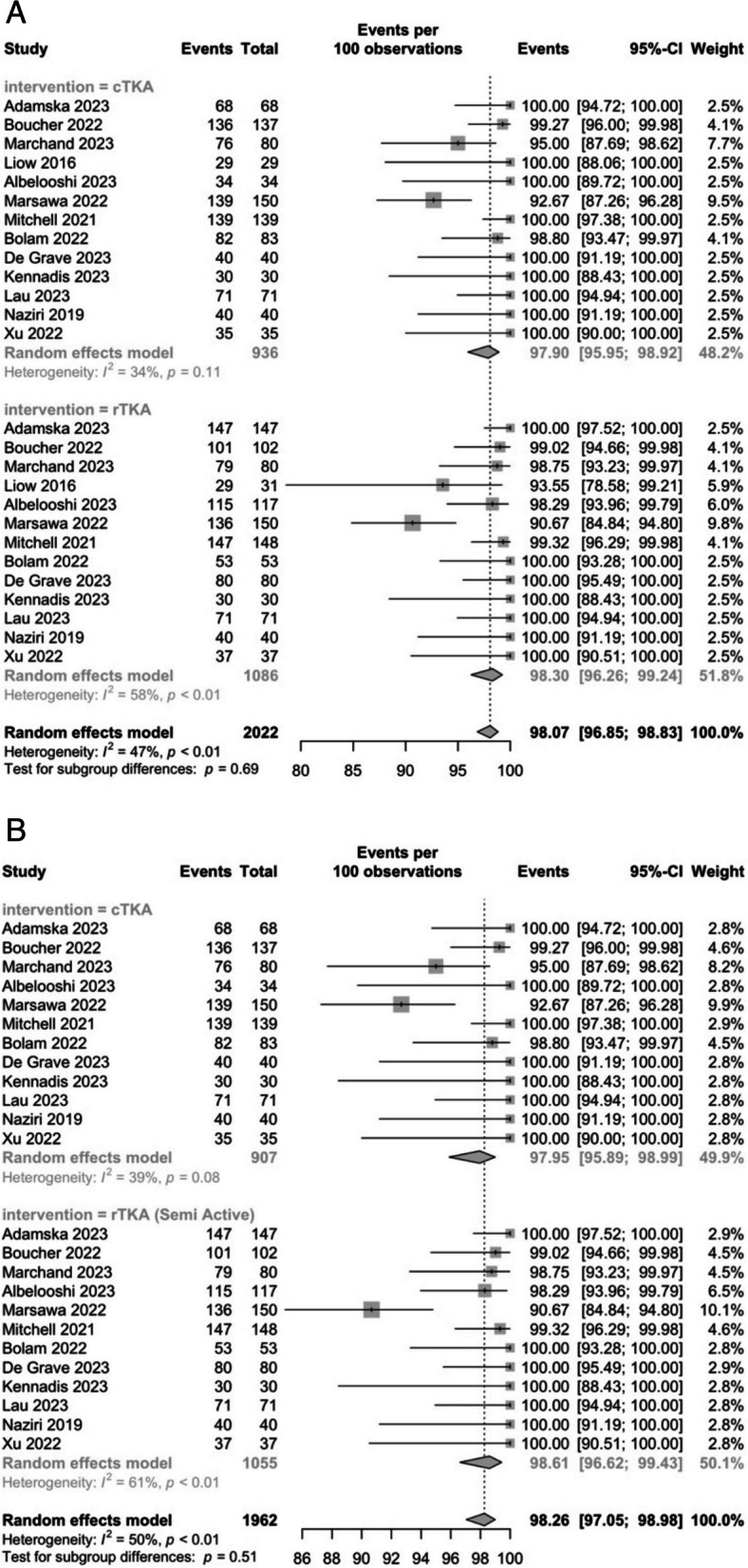
**A** Conventional and robotic TKA survivorship rates at 2-years postoperatively; (**B)** Subgrouping of rTKA group into semi-active and active, compared to cTKA

A total of 13 studies reported the 2-year survivorship of TKAs in both the conventional and robotic groups, with 936 and 1086 patients, respectively [26–29, 31, 33, 35, 37, 39–42, 44]. The pooled survival rate was 97.9% (95% CI: 96–99) in the conventional group and 98.3% (95% CI: 96.2–99.2) in the robotic group (Fig. 3A). There were no significant differences between the groups (*P* = 0.7). Heterogeneity was moderate, with an *I*^2^ value of 47%. Of the 13 studies, a semi-active robot was used in all except Liow et al. (2016). The pooled survivorship rates of semi-active robotic systems were 98.6% (95% CI: 96.6–99.4) (Fig. 3B). The robot used in Liow et al. (2016) was ROBODOC® and resulted in a survivorship rate of 93.5% (95% CI: 78.6–99.2). There were no significant differences between the robotic (semi active) group and the conventional group (*P* = 0.5) on further unpaired T-testing. Given that there was only one study in the robotic (active) group, it was not feasible to perform further statistical testing.

The reasons for revision in the conventional group were component malposition (2), hemarthrosis (1), tibial loosening (1), and joint instability & pain (13). In the robotic group: post-traumatic femur fracture (1), soft tissue impingement (2), stiffness (1), and joint instability & pain (17).

#### Five-year survival

**Fig. 4:**
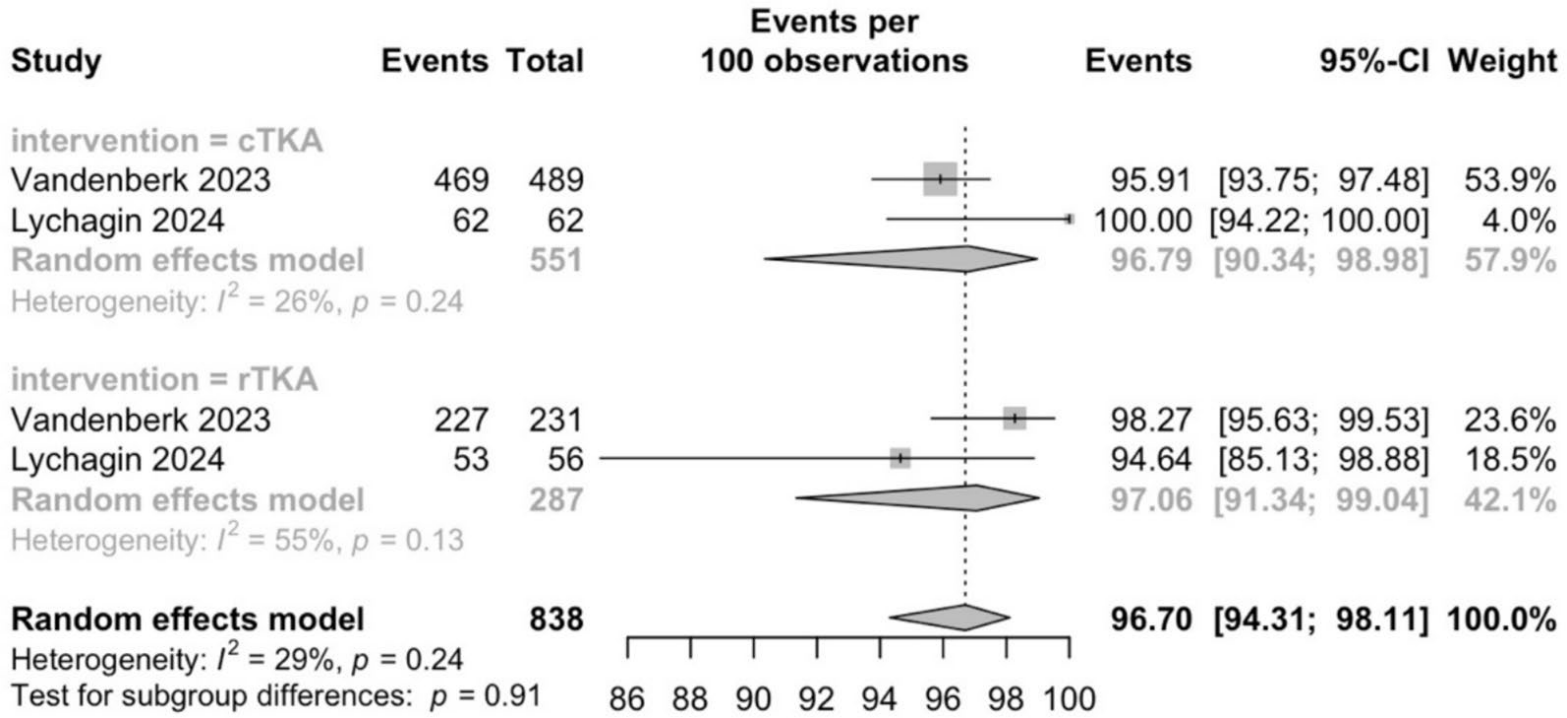
Conventional and robotic TKA survivorship rates at 5-years postoperatively

Two studies reported the 5-year survival of TKAs in both the conventional and robotic groups, with 551 and 287 patients, respectively [38, 43]. The pooled survivorship rates in the conventional group were 96.8% (95% CI: 90.3–99) and 97.1% (95% CI: 91.3–99) in the robotic group (Fig. 4). There were no significant differences between the groups (*P* = 0.9) and heterogeneity was low (*I*^2^ = 29%). Vandenberk et al. (2023) employed a semi-active robotic system in their robotic group, while Lychagin et al. (2023) used an active system. Vandenberk et al. (2023) had a greater survivorship rate of 98.3% compared to 94.6% in Lychagin et al. (2023).

The reasons for revision in the conventional group were: soft tissue impingement (4), fracture (4), DAIR (2), two-stage revision (5), open arthrolysis (1) resection of lateral osteophyte (1), perforating ACL screw (1). In the robotic group: aseptic loosening (2), infection (1), soft tissue impingement (2), fracture (2), arthroscopic plica resection (1).

#### ≥ Ten-year survival

**Fig. 5:**
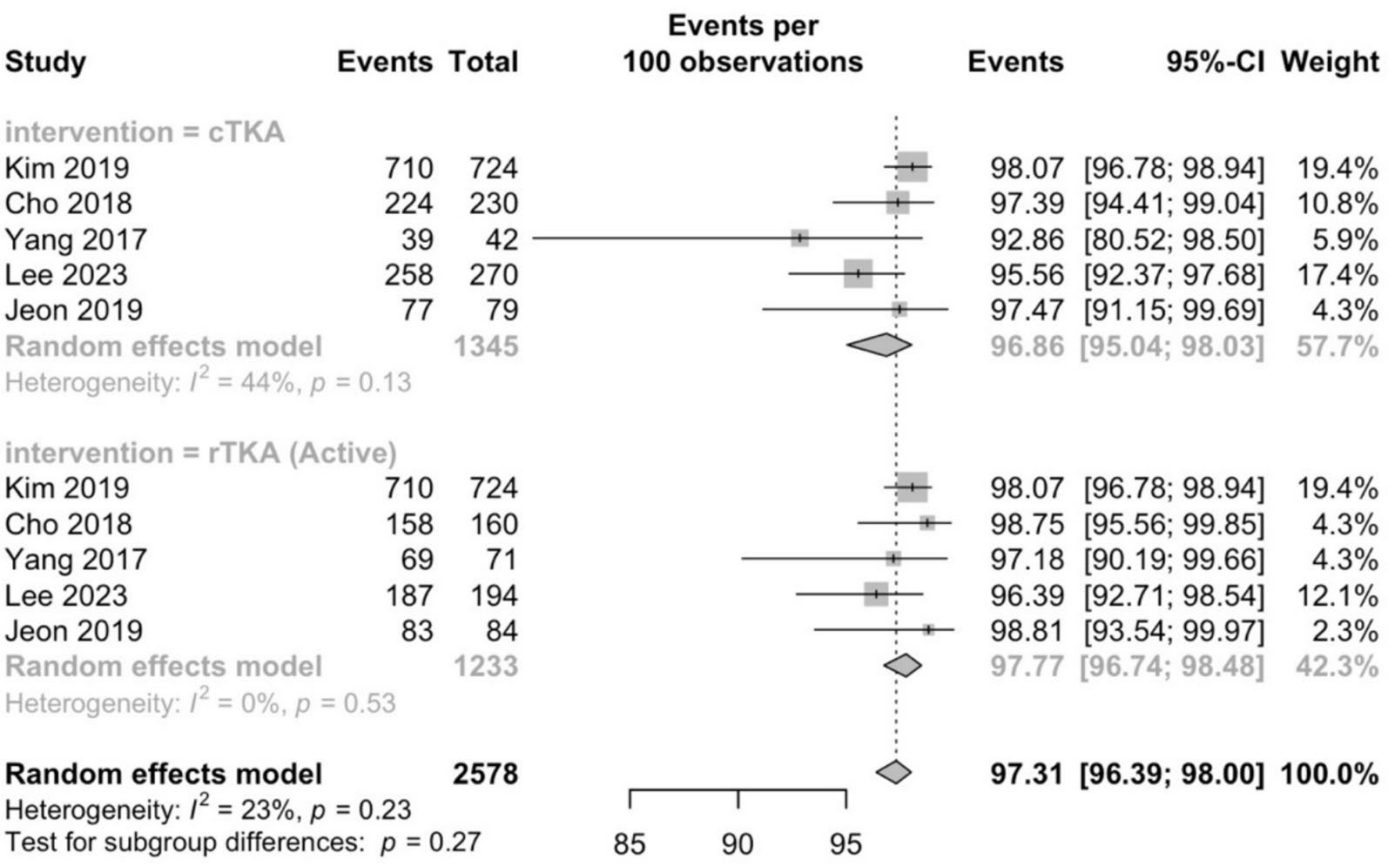
Conventional and robotic TKA survivorship rates at ≥ 10-years postoperatively

Five studies reported the survival of robotic and conventional TKAs at 10 years or more postoperatively [30, 32, 34, 36, 45]. The pooled survivorship rates in the conventional group were 96.9% (95% CI: 95–98) and 97.8% (95% CI: 96.7–98.5) in the robotic group (Fig. 5). There were no significant differences between the groups (*P* = 0.3) and the heterogeneity was low (*I*^2^ = 23%). The robotic arm of all the studies involved an active robotic system.

The reasons for revision in the conventional group were: infection (5), PE wear (4), aseptic loosening (15), instability (1), and wear of the polyethylene liner (1). In the robotic group: aseptic loosening (6) and infection (6).

### Secondary outcomes

#### Overall complication rate

**Fig. 6:**
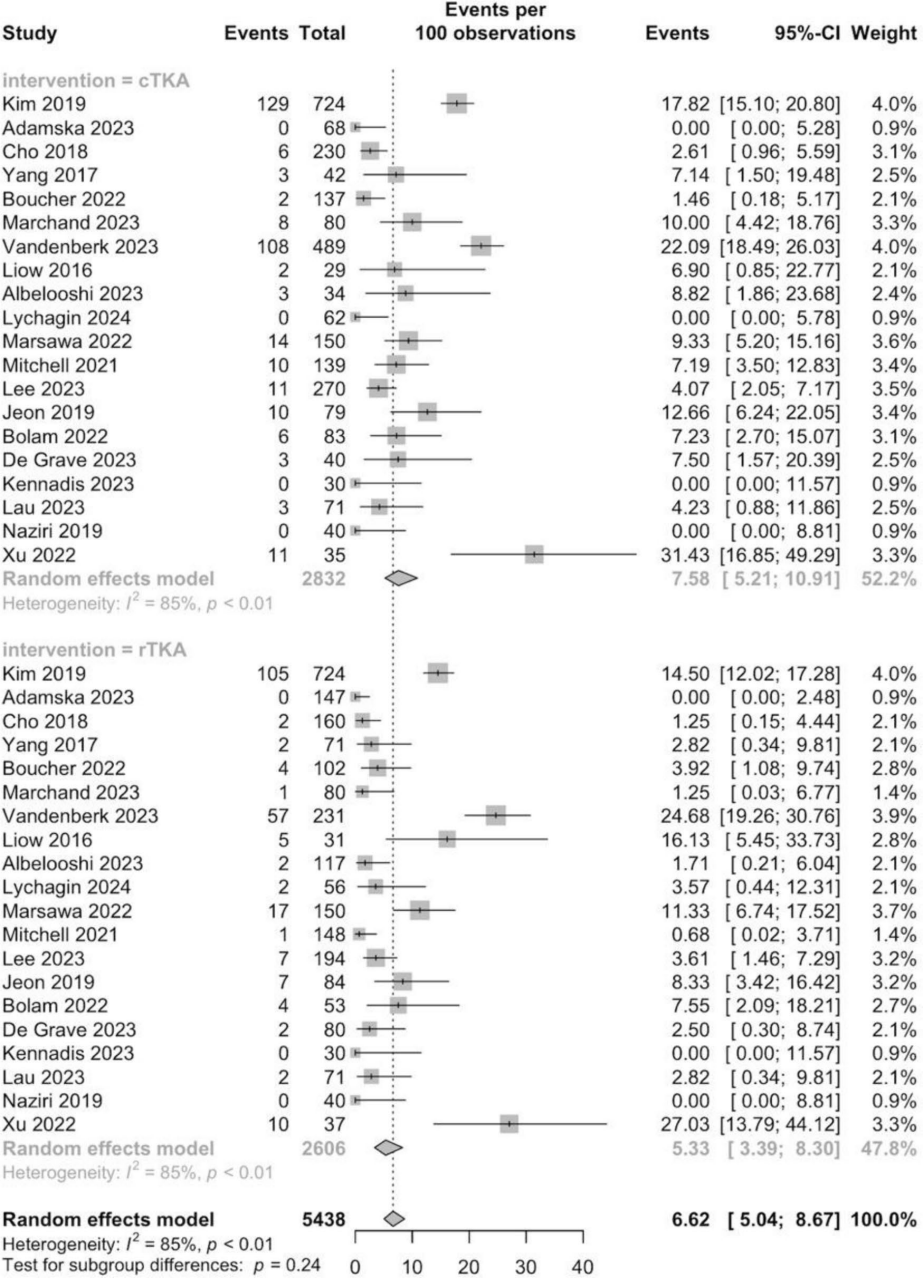
Complication rates between conventional and robotic TKA

This study analysed both medical and surgical complications. Medical complications included postoperative sepsis, infection, deep vein thrombosis or pulmonary embolism, anaemia, and myocardial infarction. Surgical complications included aseptic loosening, hemarthrosis, and arthrofibrosis. The conventional group showed a higher mean total complication rate of 7.6% (95% CI: 3.8–9.2), compared to the robotic group at 5.3% (95% CI: 2.6%–6.8), there were no significant differences between both groups (*P* = 0.2) (Fig. 6). Heterogeneity was high with an I^2^ value of 85%.

#### Infections, fractures, aseptic loosening, polyethylene wear, instability & arthrofibrosis

The incidence of specific complications was extremely low across the included studies, precluding meaningful pooled analysis. For infections, there were 27 cases among the 2,832 patients in the conventional group and 18 cases among the 2,606 patients in the robotic group. Regarding fractures, 9 cases were reported in the conventional group, while 5 fractures were observed in the robotic group. For aseptic loosening, 12 cases occurred in the conventional group compared to 7 cases in the robotic group. Polyethylene wear was reported in 4 cases in the conventional group, with no cases observed in the robotic group. Instances of instability included 12 cases in the conventional group and 14 cases in the robotic group. Finally, arthrofibrosis was documented in 14 cases in the conventional group and 8 cases in the robotic group.

#### Robotic-TKA specific complications

Reported complications specific to rTKA include pin-hole fracture, pin-related infection, iatrogenic soft tissue and bony injury, and excessive blood loss [46]. However, only 3 pin-site fractures were reported across 2,599 patients.

#### Post-operative pain score

**Fig. 7:**
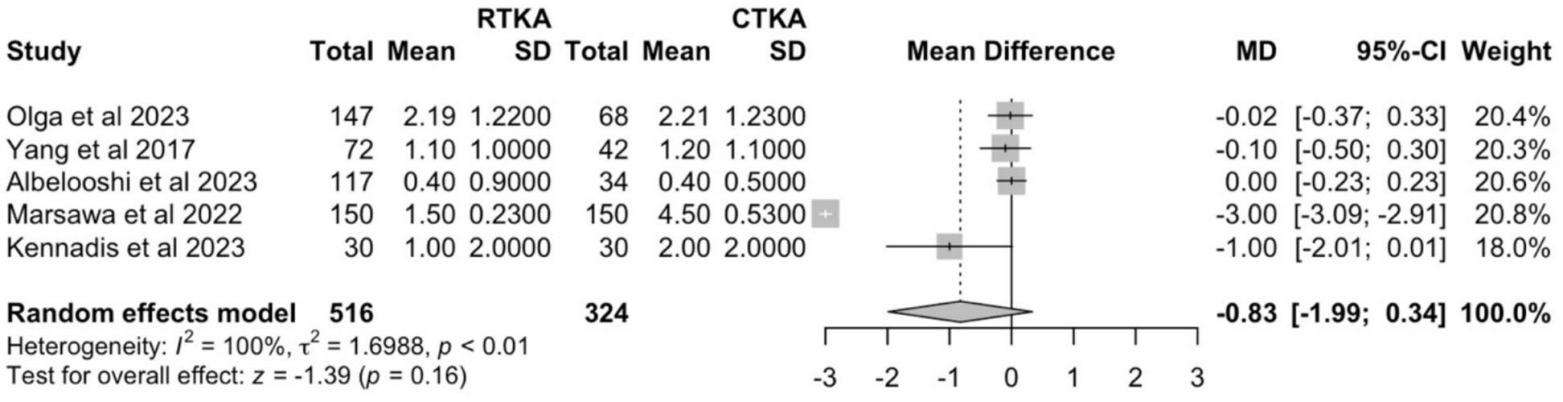
Comparison of post-operative pain scores between cTKA and rTKA at last follow-up

Five studies reported post-operative pain scores using the visual analogue scale (VAS) from 0 to 10 points (0 being no pain and 10 being the worst pain). Pain score at the latest follow-up was pooled. The cTKA group had a slightly higher post-operative pain score of 2.06 (95% CI: −0.09–4.22) compared to the rTKA group at 1.25 (95% CI: 0.69–1.81). However, this was not statistically significant (*P* = 0.16), and the high I^2^ value of 100% suggests substantial heterogeneity between studies (Fig. 7).

#### Knee society knee score

**Fig. 8:**
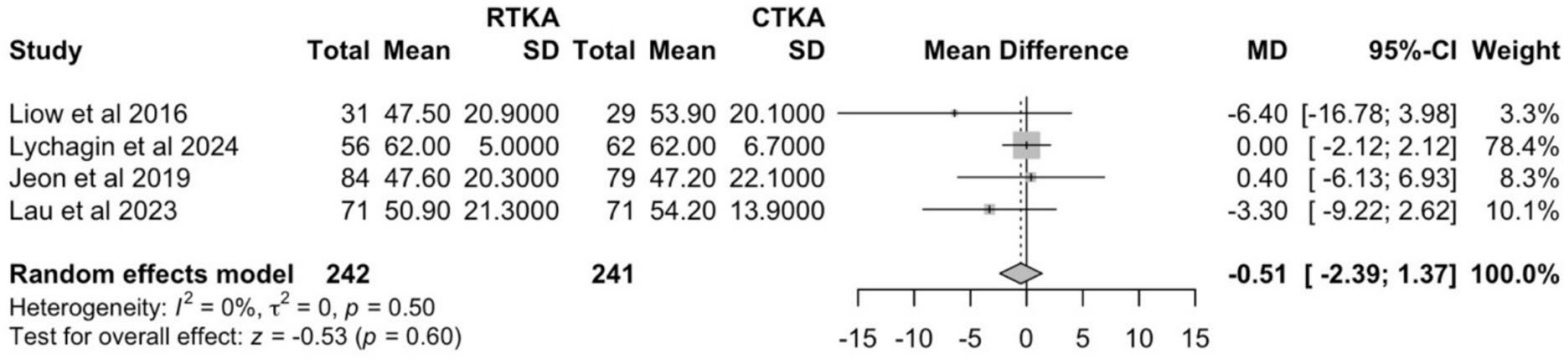
Comparison of change in KSS—knee scores between cTKA and rTKA at last follow-up

Four studies reported KSS—knee scores, and the results at the latest follow-up were pooled. Pooling of pre-operative KSS—knee scores revealed a significant difference between groups (*P* = 0.04). Hence, the Δ mean and Δ SD between pre-operative and post-operative KSS—knee scores were calculated and pooled. The cTKA group showed a slightly greater improvement in KSS—knee score of 54.6 (95% CI: 47.6–61.5) as compared to the rTKA group at 52.3 (95% CI: 43.2–61.4). However, this was not statistically significant (*P* = 0.6). The *I*^2^ value of 0% suggests there is no heterogeneity (Fig. 8).

#### Knee society function score

**Fig. 9:**
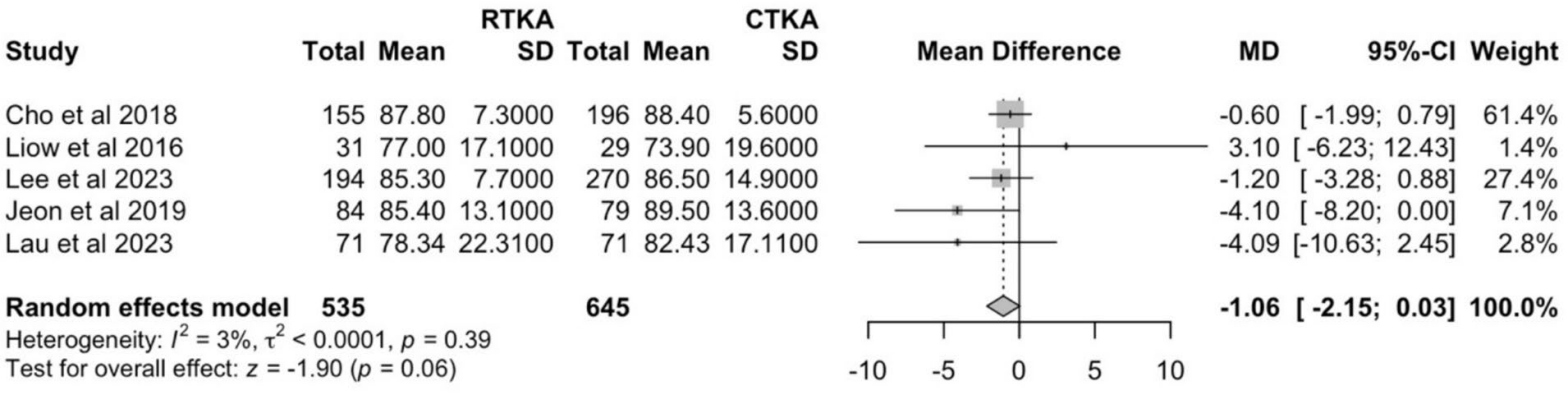
Comparison of KSS – function scores between cTKA and rTKA at last follow-up

Five studies reported KSS—function scores, and the results at the latest follow-up were pooled. Pooling of pre-operative KSS function scores showed no significant differences between groups (*P* = 0.16). Post-operatively, the cTKA group had a slightly higher KSS—function score of 85.7 (95% CI: 83.0–88.5) compared to the rTKA group at 84.2 (95% CI: 81.6–86.7). However, this was not statistically significant (*P* = 0.06). The I^2^ value of 3% suggests there is little variability between studies. (Fig. 9).

#### Post-operative WOMAC score

**Fig. 10:**
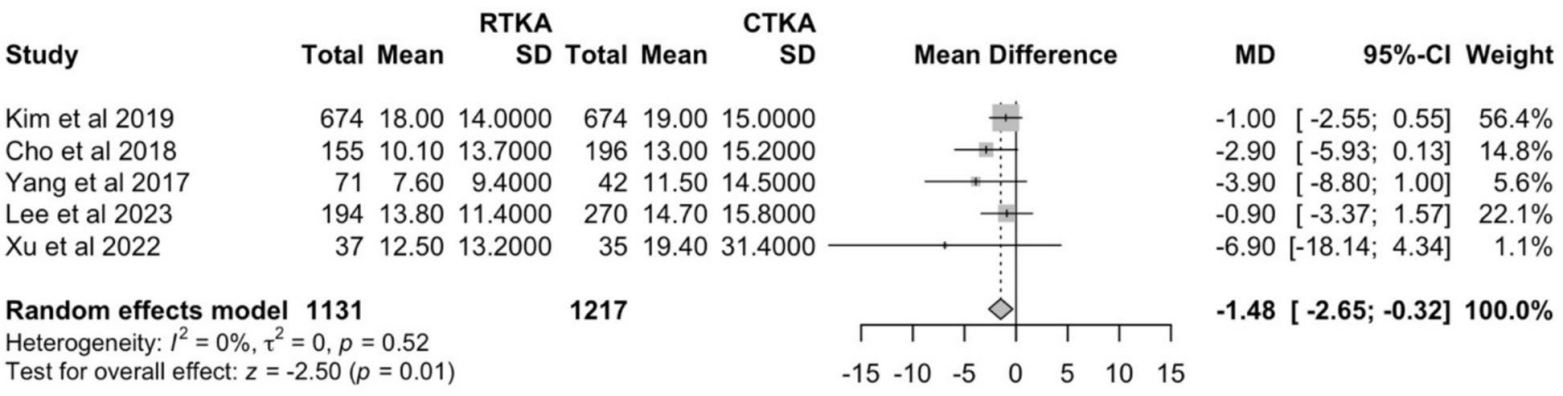
Comparison of WOMAC scores between cTKA and rTKA at last follow-up

Five studies reported post-operative WOMAC scores, and the results at the latest follow-up were pooled. The rTKA group showed a statistically significant better (*P* = 0.01) post-operative WOMAC score of 12.5 (95% CI: 8.3–16.6) as compared to the cTKA group at 15.2 (95% CI: 11.8–18.5). The *I*^2^ value of 0% suggests there is no heterogeneity. (Fig. 10).

## Discussion

The principal finding of this systematic review and meta-analysis was that for standard cases of primary knee OA, robotic-assisted TKAs were able to produce small but not statistically significant improvements to survival outcomes at short and long-term follow-up compared to conventional techniques. Results from single observations and subgrouping suggest that semi-active robotic systems do not improve short to mid-term survival compared to conventional techniques. Empirically, though, for this same period, semi-active systems have higher survivorship rates compared to active robotic systems.

Schroer and colleagues (2013) investigated the aetiologies that led to the failure of cTKAs, finding that the primary aetiologies for revisions change across the postoperative time period [47]. Up until 2-years postoperatively, the main reasons for revisions were instability, infection, and then aseptic loosening followed by arthrofibrosis. Between two and five years postoperatively, this order changes, with aseptic loosening being the primary aetiology, followed by instability and infection. After this, the main reasons for failure are aseptic loosening—making up 40% of revisions in this group—and polyethylene wear. Their findings formed the basis for the selection of these periods as outcome time points in this study. Through identifying any significance or lack thereof in survival between rTKA or cTKAs at each time point, conclusions regarding robots’ ability to prevent certain aetiologies of failure may be formed.

In the two years, no significant differences in survival were noted between the robotic and conventional groups. All but one of the studies utilised a semi-active robot, but after subgrouping to compare the semi-active rTKA group to cTKA, semi-active rTKA exhibited greater but still non-statistically significant improved survival compared to cTKA. Liow et al. (2016), who used ROBODOC®, presented relatively lower survivorship rates compared to most of the others who had used a semi-active system [37]. Interestingly, the main reasons reported for revision in both groups were instability and pain, reflecting Schroer’s work. Early instability has been attributed to component malalignment and imbalance in the flexion and extension gaps [48]. Held et al. (2021) suggested that in terms of balance, robotics was advantageous over conventional in full flexion but not in extension and midflexion [49].

Survival was also comparable between both groups at the 2–5-year postoperative timeframe. The active robot system in this period also resulted in lower survivorship rates than the semi-active robot. Active systems have been reported to have higher complication rates, in particular, soft tissue injury and fractures, which may have contributed to the general higher revision [50, 51]. Interestingly, at 10 years and longer, the mean survival rate of active robotic systems was greater than the earlier timeframes. The study team hypothesises that if patients avoid the earlier complications of instability, infection, or technical complications, rTKA may be able to reduce aseptic loosening and polyethylene wear compared to conventional methods. Indeed, aseptic loosening and polyethylene wear were the reasons for nearly 80% of revisions in the conventional group but only accounted for half in the robotic group in this study.

Generally, survival rates were high in both robotic and conventional groups, and interestingly, survival did not decline steeply with increased follow-up duration. At early (up to 2 years) follow-up, both groups had a survival of approximately 98%, and this remained comparable even at 10 years follow-up. Few studies in literature individually describe survival trends of robotic and conventional TKA at various follow-up time points, likely owing to the short duration of time semi-active robotic systems have been in use and the inconsistent uptake of active systems globally. As time progresses, further research should explore survival outcomes at different time points. A registry study by Ofa and colleagues (2020) shared similar high rates at 97% at short-term follow-up [52], and longer term follow-up studies by Kim, Cho, and Yang et al. were in agreement with respect to survival rates, at approximately 97%–98%[30, 36, 45].

The lack of a specific meta-analysis focusing on the survival outcomes between conventional and robotic TKA prompted the current systematic review and meta-analysis. Functional and radiologic outcomes have been well-reported across multiple meta-analyses, but whether such improvement translated to better survival is unclear. The subgrouping relating to semi-active and active systems was also novel to this meta-analysis, although a direct comparison between semi-active and active systems was not possible due to the paucity of data. This study established that rTKA, in general, may cause a small but not significant improvement in survival. At short to medium-term durations, active systems were identified to have lower survival rates than semi-active systems.

The non-statistical significance in short-term survival outcomes between robotic and conventional methods had been postulated to similar rates of early instability caused by imbalance in flexion-and-extension gaps. Instability is a major factor for early TKA revision, and robotic technology alone may not necessarily help to resolve this issue. While robotics assist with the gap balancing process, symmetry in flexion and extension gaps does not always result in a balanced knee [53, 54]. Restoring native knee anatomy and kinematics should be one of the considerations in performing TKAs.

Clinical outcomes are an important factor in assessing functional recovery. We found that KSS and pain scores were comparable between conventional and robotic groups, but rTKA was associated with a greater increase in postoperative WOMAC scores at the final follow-up. The WOMAC score encompasses pain, stiffness, and physical function; compared to the KSS scores that account for more clinical and objective measures such as range of motion, flexion contractures, alignment, and stability on top of pain and physical function. The discrepancy in these clinical outcomes may suggest that better implant positioning and accuracy lead to improved subjective outcomes despite equivocal clinical evaluation in robotic TKA compared to conventional methods [10–13]. Arguably, there is mixed evidence over rTKA’s significance in improving various functional outcome scores, such as in Zhang et al. (2022), where KSS and WOMAC were both significantly improved, whilst Argawal et al. (2020) shows an improvement in WOMAC but not KSS [13, 55]. This could be influenced by differing study selection and follow-up durations, such as in our present study. Furthermore, due to the limited follow-up duration in the present study, its impact on survivorship outcomes may not have been apparent. Future research focusing on long-term survival outcomes will be useful in evaluating if an improvement in functional outcomes translates to better survival outcomes in the setting of rTKA.

Separately, an important consideration in comparisons between robotic and conventional TKAs is cost-effectiveness. Cost-effectiveness remains an understudied aspect of rTKAs. Typically, this has been measured using cost per quality-adjusted life-year (QALYs). While this study does not address this, present evidence is conflicting, with Zhang et al. (2023) finding rTKA not cost-effective—an overall gain in QALYs of 0.03 for each patient was undermined by an incremental cost of $128,526 Singapore dollars per QALY [56]. Rajan et al. (2022) demonstrated similar modest improvement in QALYs, with 13.55 QALYs after rTKA compared to 13.29 QALYs in cTKA [57]. Interestingly, they found a stark decrease in cost per QALYs when comparing low-volume, mid-volume, and high-volume centres—$256,055/QALY (low volume), $15,685/QALY (mid volume), and $2,331/QALY (high volume) [57]. Taken together, rTKA may be cost-effective in high-volume settings, however, the number of cases required to make the technology cost-effective is yet to be determined. From the patient perspective, Alton and colleagues (2023) found the procedure to be overall cost-neutral, with cost savings from faster home discharge and decreased 90-day readmission rates offsetting the increased cost [58]. Future research should be directed at high-quality evidence regarding cost-analysis of rTKAs compared to cTKAs.

The current review exhibited several strengths. First, this study represented the first systematic review and meta-analysis to have compared survival outcomes of cTKA and rTKA. This focus of the meta-analysis allowed for findings that will be useful in the patient-surgeon conversation regarding prostheses survival and reasons for failure at specific time points. Second, identification and further subgrouping of the type of robotic systems—semi-active or active—which have different mechanisms of operation, reduced the heterogeneity and allowed for system-specific comparisons to conventional methods. Across primary outcomes, the studies displayed low heterogeneity and no significant publication bias, which further lends confidence to the conclusions drawn. Third, the systematic review and meta-analysis pooled the reasons for TKA revision across both groups, which enabled the identification of major aetiologies for failure at different time points postoperatively.

While limited data precludes us from obtaining meaningful results when subgrouping among different population demographics, the mean age of patients in our study was between 65 and 70 years, with an average BMI of 29 kg/m^2^. These have been reported to be the typical characteristics of patients who undergo TKA [59–61]. Moreover, it has been shown that lower BMI is associated with improved post-TKA outcomes [62–64]. For non-obese patients, it may be inferred that clinical outcomes and survival will be similarly high, although definitive studies are required to conclude this. Patients in our group were mostly female, but gender is not a significant predictive factor influencing post-TKA outcomes. Nonetheless, given the present patient demographic, caution ought to be taken when generalising results to younger cohorts undergoing TKA.

The present review includes a majority of non-randomized observational studies, which may introduce heterogeneity due to variations in patient demographics, degree of OA, alignment philosophy utilised, and surgeon experience. While heterogeneity was low in primary outcomes, it was reported to be high in complication rates, with an *I*^2^ of 85%. This may be attributed to definitions of complications, as some studies may include minor adverse events while others focus only on major complications. Additionally, variations in institutional perioperative protocols could further contribute to this inconsistency. Given this high heterogeneity, the interpretation of complication outcomes should be approached with caution. Future research would benefit from standardized complication reporting and subgroup analyses to better understand the factors influencing these variations.

The current review also faced several limitations. First, the level of evidence of the review is limited by the presence of adequately powered trials in the current literature. Indeed, while the studies included in this systematic review and meta-analysis were RCTs, prospective comparative studies and cohort studies, the topic could benefit from more higher-powered RCTs to further confirm or refute the conclusions drawn.

Second, while subgrouping to compare semi-active robotic and conventional TKA at two years, along with active robotic and conventional TKA at ten years or more was possible, statistical comparisons between semi-active and active systems were not possible. Semi-active systems have only been in use since 2017, limiting the long-term survival data available.

Third, all the studies in the ten-years-or-more postoperative group were performed in South Korea, possibly introducing selection bias. The studies by Cho et al. (2019)[30], Lee et al. (2023) [36], and Yang et al. (2019) [45] reported different study protocols, but study teams were similar. Therefore, introducing the possibility of overlap of patients within these included studies. The decision was made, however, to still include both papers in the review as separate papers because of the different study protocols, patient demographics, and results reported. To address the potential overlap, a sensitivity analysis was performed, which can be found in the supplementary information. This analysis excluded studies with overlapping authorship to assess the robustness of the results. The findings remained consistent, with no significant differences observed, lending support that the studies were suitable for inclusion.

Robotic TKA has enabled surgeons to precisely and accurately position their bony cuts, implants, and gap measurements [65–68]. This can allow surgeons to achieve their surgical goals in alignment with their preferred philosophy. As component positioning and limb alignment are associated with improved outcomes post-TKA [66, 68, 69], further research to determine the most effective alignment philosophy in different patient groups may enhance clinical outcomes. Incorporation of artificial intelligence (AI) and machine-learning (ML) algorithms has also been suggested to improve the decision-making process and subsequent outcomes [70]. With such improvements and more, long-term revision rates may be improved and hence improve cost-parity with cTKAs [71].

## Conclusion

Robotic TKAs produce small but not statistically significant improvements to survival compared to conventional methods at short and long-term follow-up. However, adequate data are lacking to make definitive conclusions regarding comparisons between semi-active and active robotic systems. Furthermore, while rTKA demonstrated improved functional outcomes in terms of the WOMAC score, impacts on overall complication rates, post-operative pain score, and KSS were non-statistically significant. Further high-quality studies with longer follow-up periods are required to establish whether functional advantages translate into better survival outcomes.

## Data Availability

All data in the manuscript will be made available on reasonable request.

## Abbreviations

cTKA: Conventional total knee arthroplasty
OA: Osteoarthritis
PRISMA: Preferred Reporting Items for Systematic reviews and Meta-analyses Statement
RCT: Randomized Controlled Trial
rTKA: Robotic total knee arthroplasty
TKA: Total knee arthroplasty
KSS: Knee Society Score
WOMAC: Western Ontario and McMaster Universities Osteoarthritis Index
SD: Standard Deviation
